# Efficacy of the Treatment of Elderly Trauma Patients Requiring Intensive Care

**DOI:** 10.1155/2018/2137658

**Published:** 2018-12-30

**Authors:** Kiyohiro Oshima, Masato Murata, Makoto Aoki, Jun Nakajima, Yusuke Sawada, Yuta Isshiki, Yumi Ichikawa, Kazunori Fukushima, Shuichi Hagiwara, Hiroshi Hinohara

**Affiliations:** ^1^Department of Emergency Medicine, Gunma University Graduate School of Medicine, Japan; ^2^Intensive Care Unit, Gunma University Hospital, Japan

## Abstract

**Purpose:**

To evaluate the effectiveness of intensive care for the elderly trauma patients aged 80 years and older.

**Methods:**

Trauma patients admitted to the intensive care unit (ICU) through the emergency room (ER) at our hospital between January 2013 and December 2016 were analyzed. Patients were divided into two groups: patients aged 80 and older (group E) and <80 years old (group Y). Clinical courses and the total treatment costs were compared between the two groups. Data are shown as median (interquartile range).

**Results:**

A hundred and seven trauma patients were included in the study. There were 26 patients in group E and 81 patients in group Y. There was no significant difference in Injury Severity Score (ISS) (group E, 19 (13, 32); group Y, 17 (14, 25); p=0.708); however, the probability of survival (Ps) was significantly lower in group E (group E, 0.895 (0.757, 0.950); group Y, 0.955 (0.878, 0.986); p=0.004). The duration of ICU stay (days) was significantly longer in group E (10 (5, 23)) than in group Y (4 (3, 9); p=0.001), and the total hospital stay (days) was longer in group E (33 (13, 57)) than in group Y (22 (12, 42); p=0.179). The hospital mortality was higher in group E (11.5%) than in group Y (6.2%) without a significant difference (p=0.365). The total treatment costs were significantly higher in group E ($23,558 (12,456, 42,790) with $1 = ¥110.57) than in group Y ($16,538 (7,412, 25,422); p=0.023).

**Conclusions:**

Elderly trauma patients require longer-term treatment including ICU stay and greater cost with higher hospital mortality compared with young trauma patients.

## 1. Introduction

Life expectancy in developed countries including Japan has increased considerably, with the population living longer while aging rapidly during the last few decades [1, 2]. The World Factbook published by the US Central Intelligence Agency in 2016 reports that an average life span in developed countries is almost 80 years and older such as 80.80 years in the United Kingdom, 80.00 years in the United States, and 85.30 years in Japan [3]. Accordingly, emergency ambulance dispatches to assist elderly people have increased recently as the Japanese population ages. The 2016 annual report of the Fire and Disaster Management Agency in Japan indicated that 57% of emergency ambulance dispatches were to assist people 65 years old and older [4].

Elderly patients admitted to ICUs have a high risk of death [5], and the effectiveness of the intensive care for elderly patients is controversial. In addition, Heyland DK et al. [6] reported that more than 70% of seriously ill hospitalized elderly patients do not take advantage of their ability to discuss their preferences with their healthcare providers. Unfortunately, no guidelines have been established in regards to the treatment of elderly patients.

Trauma is a leading cause of death in the elderly population of developed countries [7, 8]. Geriatric trauma patients show greater morbidity and mortality than younger trauma patients due to decreased physiological reserve, frailty, and preinjury comorbidities [9–11]. Demetriades et al. reported that age is an important factor in predicting the risk of fatal outcomes after traumatic injuries [12]. While the impact of age in trauma patient outcomes is clear, few studies have examined the cost-effectiveness of the treatment of elderly trauma patients.

We regarded a criterion of an average life span in developed countries as 80 years old and hypothesize that the length of hospital stay is longer, hospital mortality is poorer, and the total treatment cost is more expensive in cases of elderly trauma patients (80 years and older) admitted to the intensive care unit (ICU) through the emergency room (ER) than that of younger trauma patients. The purpose of this study was to evaluate the effectiveness of intensive care for the elderly trauma patient aged 80 years and older based on not only outcome, but also cost-effectiveness.

## 2. Patients and Methods

The protocol of this retrospective study was approved by the research ethics board of the Gunma University Hospital without the need for informed consent (#2016-044), and the enforcement of this study was announced on the homepage of our university.

Trauma patients who were admitted to our ICU through ER in our hospital between January 2013 and December 2016 were examined. Patients admitted ICU after emergency operation and/or transcatheter arterial embolization through ER were also included. E Exclusion criteria were patients <18 years old, patients removed from ICU within 24 hours, and patients with terminal stage malignant disease or liver cirrhosis. Patients were divided into two groups: patients aged 80 years and older (group E) and patients less than 80 years old (group Y). We compared Sequential Organ Failure Assessment (SOFA) score, the abbreviated injury scale (AIS) of six anatomical regions (head/neck, face, chest, abdomen/pelvis, extremities, and skin/general), the Injury Severity Score (ISS, the highest AIS scores in the three most severely injured regions are squared and summed to obtain the ISS), and the probability of survival (Ps) at arrival between the two groups. The duration of stay in ICU, the total hospital days, the hospital mortality, and the total treatment costs were also compared {In Japan, Diagnosis Procedure Combination (DPC) system was started from April 2003. DPC system is an inclusion payment system based on the diagnosis group classification for the hospital treatment per day for an acute phase. Approximately 83% of hospitals corresponding to the acute phase patients in Japan introduce this system as of April, 2018. Our hospital also use this system to calculate the treatment cost}.

### 2.1. Statistical Analysis

In our hospital, there were 160 patients admitted ICU through ER in 2013 (≧80 years old: 32, <80 years old: 128, respectively). Sample size was calculated as 105 with 0.8 of power, 5% of significance level, and 0.5 of effect size. Data were shown as real number or median (Q1, Q3) and the differences were analyzed with Mann-Whitney U test, chi-square test, or Fisher's exact test. A p value of less than 0.05 was considered statistically significant. The IBM SPSS Statistics 22 was used for the statistical analysis.

## 3. Results

**Figure 1:**
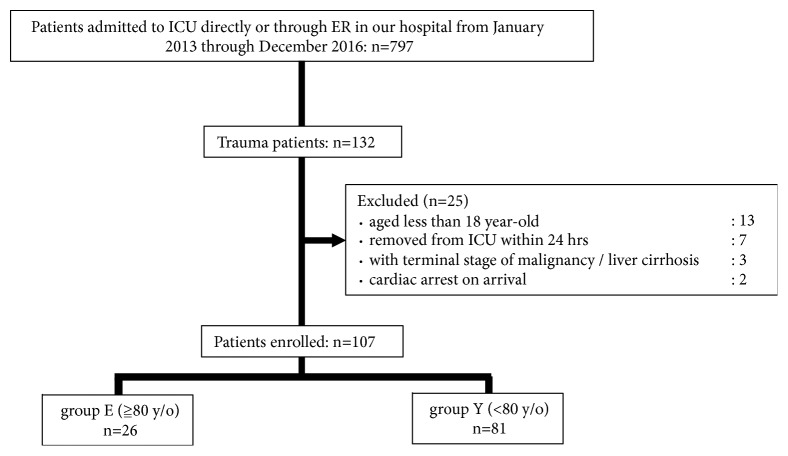


From January 2013 to December 2016, 797 patients were admitted to ICU through the ER. Of those patients, 107 trauma patients were included in the study (Figure 1). There were no problems in activities of daily living in all 107 patients before they were injured and there were no patients with Do Not Attempt CPR order and no patients who were transferred to convalescent hospital in those 107 patients.

The causes of trauma were traffic accident in 57 patients, fall in 33 patients, and other causes in 17 patients. There were 101 patients with blunt trauma and 6 with penetrating trauma. The median age of all 107 patients (79 male, 28 female) was 65 (45, 79) years old. The medians of ISS and Ps were 18 (14, 25) and 0.943 (0.801, 0.977), respectively. Median duration of ICU stay and hospital stay were 5 (3, 10) days and 24 (12, 46) days, respectively. Hospital mortality was 7.5% (8/107), and the total medical costs ($1 = ¥110.57) were $17,107 (7,868, 32,378) for all 107 patients.

There were 26 patients in group E (age, 83 (81, 85) years, ranging 19 ~ 79 years) and 81 in group Y (age, 58 (36, 68) years, ranging 80 ~ 89 years), respectively. The male/female ratio, comorbidities and causes of trauma in both groups are shown in Table 1. There were no significant differences in comorbidities and the rate of traffic accident and fall between the two groups. However, other causes, including penetrating trauma in 6 patients, ski/snowboard in 3 patients, slip in 3 patients, being caught by machine in 2 patients, being blown due to tree in 2 patients, and equestrian injury in 1 patient, were significantly higher in group Y than in group E. There was no significant difference in SOFA score at arrival to our hospital between the two groups. The AISs of chest and abdomen were significantly higher in group E than in group Y (Table 1).

**Figure 2:**
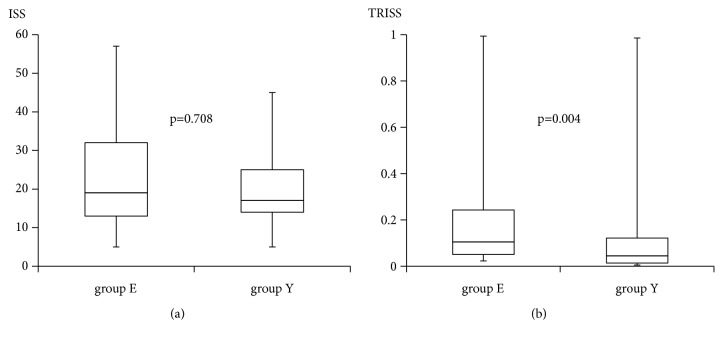


There was no significant difference in ISS between the two groups (group E, 19 (13, 32); group Y, 17 (14, 25); p=0.708) (Figure 2(a)); however, Ps was significantly lower in group E (0.895 (0.757, 0.950)) than in group Y (0.955 (0.878, 0.986); p=0.004) (Figure 2(b)).

**Figure 3:**
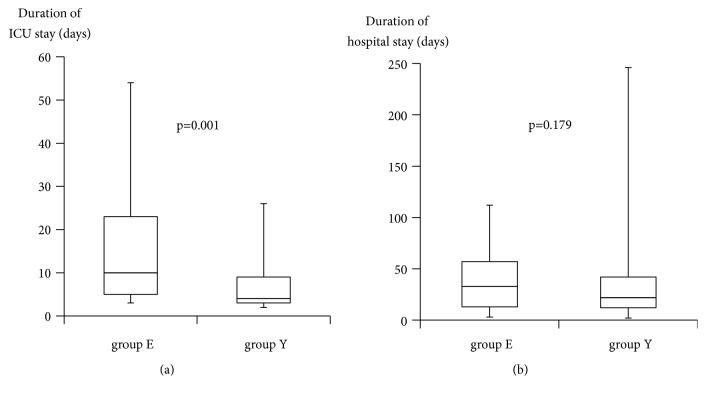


The duration of ICU stay was significantly longer in group E (10 (5, 23) days) than in group Y (4 (3, 9) days; p=0.001) (Figure 3(a)), and the total hospital stay was longer in group E (33 (13, 57) days) than in group Y (22 (12, 42) days) without significant difference (p=0.179) (Figure 3(b)).

**Figure 4:**
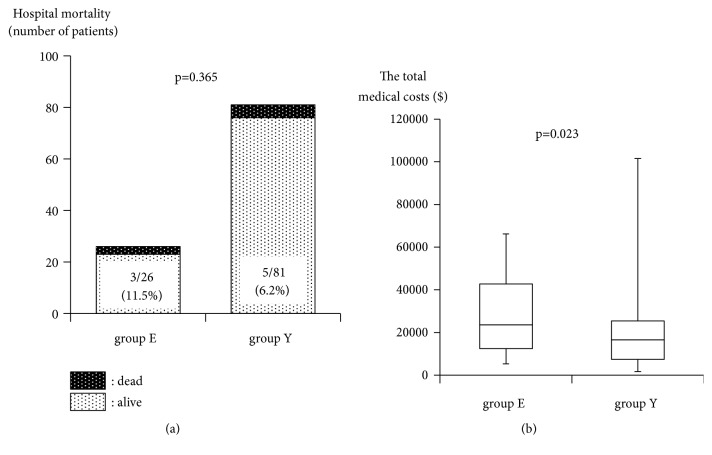


The hospital mortality was higher in group E (11.5%) than in group Y (6.2%) without a significant difference (p=0.365, Figure 4(a)). The total treatment costs were significantly higher in group E ($23,558 (12,456, 42,790)) than in group Y ($16,538 (7,412, 25,422); p=0.023) (Figure 4(b)).

## 4. Discussion

In this study, the median ISS, which reflects trauma severity, was 18 in all patients, and >15 in both groups. In general, severe trauma is defined as ISS>15 [13, 14], therefore, the subjects in this study were patients with severe trauma. The causes of trauma in group Y were not only traffic accident and fall, but also other causes, while causes of trauma in group E were only traffic accident and fall. There was no significant difference in ISS between the groups; however, Ps was significantly lower in group E than in the group Y. In addition, AISs of the chest and abdomen were significantly higher in group E than in group Y. The value of Ps is affected whether the age is 55 years or less. In this study, patients were divided into two groups based on the age of 80 years. Group Y included 44 patients (54.3%) 56 years old and older. Therefore, we observed little effect of age on Ps between the two groups. Our results suggest that the influence of trauma on general status such as consciousness, blood pressure, and/or respiratory rate is more severe in elderly patients compared with younger patients in cases of trauma of similar degree though those were not reflected to SOFA score. It is well known that there is a decline in physiologic reserve with aging [15, 16], and our results are consistent with those findings.

The duration of ICU stay was significantly longer in the elderly group, while the length of hospital stay was also longer in the elderly group, but the difference was not significant. The hospital mortality was higher in the elderly group, and, finally, the total medical costs increased in the elderly patient group compared with the younger patient group. These results suggest that elderly trauma patients are more likely to become more severe, and, therefore, they require long-term ICU management. The longer duration of ICU stay results in increased medical costs, and the hospital mortality tended to be still higher in elderly trauma patients even if intensive care was provided.

Studies have reported that elderly trauma patients show higher mortality rates and worse long-term outcomes than younger patients [17, 18] due to pre-existing comorbidities [19], decreased physiologic reserve [20], and undertriage [21]. Hashmi et al. [18] reported that patients older than 74 years with traumatic injuries are at a higher risk for mortality than the younger geriatric group. On the other hand, Chelluri et al. [22] reviewed the literature on outcomes of intensive care for the elderly with regard to ICU utilization, mortality, hospital costs and charges, and quality of life after intensive care. They concluded that age alone is not an acceptable predictor of critical illness with regard to mortality and quality of life of survivors. In addition, Marik [23] insisted that functional elderly patients have a favorable “long-term” outcome after ICU admission, and age alone should not be used in making intensive care unit triage decisions. Previously, we studied patients aged 90 years and older, including trauma patients, who were admitted to our department through the ER, and 77.6% of those patients were discharged from our hospital [24]. Additionally, we examined the effectiveness of cardiopulmonary resuscitation (CPR) in patients who were 80 years of age and older and presenting with cardiopulmonary arrest on arrival (CPAOA). Results suggested that we should not endorse a policy that recommends not performing CPR based solely on the age of the patient because even patients aged 80 years and older can be resuscitated to spontaneous circulation [25]. As described above, the opinion regarding the treatment for elderly patients has not established yet and is still controversial. Our results suggest that ICU treatment may be ineffective and the effect of treatment that we expect can't be obtained even though greater treatment costs are incurred in the elderly trauma patients. For the implementation of substantial medical care for elderly trauma patients, guidance in identifying techniques and practices with proven capacity to improve outcomes should be required [26]. Griffiths and Kumar suggested that a review of triage criteria is necessary to improve the identification of patients ≥ 60 years old with severe injuries [27]. In fact, Cook et al. reported the usefulness of a prognostic tool for geriatric mortality after injury called the Geriatric Trauma Outcome Score (GTOS) [20]. The establishment of a practice system specialized in elderly trauma patients will be necessary in the future.

## 5. Limitations

This study was retrospective and performed at only one institution, and the number of patients was not very large. It may be the influence of the number of patients that there was no significant difference in hospital mortality between the two groups even though that in group E was approximately 2 times in group Y. Our institution is not a trauma center specialized in trauma management. We evaluated only clinical data, and an examination of the detailed mechanism of our results has not been performed. In addition, the evaluation of long-term prognosis was not performed at all. A multicenter study is necessary to further clarify the appropriate treatment of elderly trauma patients.

## 6. Conclusion

Elderly trauma patients require longer-term treatment including ICU stay and increased costs; however, hospital mortality may be finally higher in elderly trauma patients compared to young trauma patients.

## Figures and Tables

**Table 1:** Comparison between the two groups.

	group E (n=26)	group Y (n=81)	p value
Age	83 (81, 85)	58 (36, 68)	<0.001

Male/Female	61/20	18/8	0.376

Comorbidities			
Diabetes Mellitus	5 (19.2%)	11 (13.6%)	0.482
Hypertension	7 (26.9%)	11 (13.6%)	0.114
Heart disease	5 (19.2%)	6 (7.4%)	0.084
Respiratory disease	0	2 (2.5%)	0.419
Hepatitis	1 (3.8%)	3 (3.7%)	0.973
Maintenance dialysis	0	1 (1.2%)	0.569
Oral anticoagulants	5 (19.2%)	7 (8.4%)	0.137

The causes of trauma			
Traffic accident	14 (54%)	43 (53%)	0.946
Fall	12 (46%)	21 (26%)	0.052
Others	0	17 (21%)	0.012

SOFA score	3 (2, 4)	3 (1, 4)	0.260

AIS			
Head/Neck	0 (0, 3)	1 (0, 4)	0.238
Face	0 (0, 0)	0 (0, 1)	0.136
Chest	3 (1, 4)	0 (0, 3)	0.015
Abdomen/Pelvis	2 (0, 3)	0 (0, 2)	0.023
Extremities	0 (0, 2)	1 (0, 2)	0.238
Skin/General	0 (0, 0)	0 (0, 0)	0.740

## Data Availability

Our data in this manuscript were obtained from trauma patients transferred to Gunma University Hospital between January 2013 and December 2016. If you ask the authors to show data regarding this manuscript, they can show them.
